# Association between Brain and Plasma Glutamine Levels in Healthy Young Subjects Investigated by MRS and LC/MS

**DOI:** 10.3390/nu11071649

**Published:** 2019-07-19

**Authors:** Yuhei Takado, Naoto Sato, Yuta Kanbe, Moyoko Tomiyasu, Lijing Xin, Jamie Near, Kohki Yoshikawa, Naruhiko Sahara, Tatsuya Higashi, Tetsuya Suhara, Makoto Higuchi, Takayuki Obata

**Affiliations:** 1 National Institute of Radiological Sciences, National Institutes for Quantum and Radiological Science and Technology, Chiba 263-8555, Japan; 2 Animal Imaging and Technology Core (AIT), Center for Biomedical Imaging (CIBM), Ecole Polytechnique Fédérale de Lausanne, 1015 Lausanne, Switzerland; 3 Douglas Mental Health University Institute and Department of Psychiatry, McGill University, Montreal, QC H4H 1R3, Canada; 4 Department of Radiological Sciences, Faculty of Health Sciences, Komazawa University, Tokyo 154-8525, Japan

**Keywords:** magnetic resonance spectroscopy, glutamine, glutamate, LC/MS, posterior cingulate gyrus, cerebellum

## Abstract

Both glutamine (Gln) and glutamate (Glu) are known to exist in plasma and brain. However, despite the assumed relationship between brain and plasma, no studies have clarified the association between them. Proton magnetic resonance spectroscopy (MRS) was sequentially performed twice, with a 60-min interval, on 10 males and 10 females using a 3T scanner. Blood samples for liquid chromatography–mass spectrometry (LC/MS) to measure Gln and Glu concentrations in plasma were collected during the time interval between the two MRS sessions. MRS voxels of interest were localized at the posterior cingulate cortex (PCC) and cerebellum (Cbll) and measured by the SPECIAL sequence. Spearman’s correlation coefficient was used to examine the association between brain and plasma metabolites. The Gln concentrations in PCC (mean of two measurements) were positively correlated with Gln concentrations in plasma (*p* < 0.01, *r* = 0.72). However, the Glu concentrations in the two regions were not correlated with those in plasma. Consideration of the different dynamics of Gln and Glu between plasma and brain is crucial when addressing the pathomechanism and therapeutic strategies for brain disorders such as Alzheimer’s disease and hepatic encephalopathy.

## 1. Introduction

Glutamine (Gln) and glutamate (Glu) are abundantly present and have important roles in brain and plasma [1]. The concentrations of both molecules are often altered in brain disorders such as hepatic encephalopathy, neurodegenerative disorders and psychiatric disorders [2,3,4,5]. However, the mechanism behind this phenomenon remains unclear. 

Gln is one of the most abundant amino acids in blood [6], and the effect of its supplementation on clinical symptoms of neurodegenerative diseases has been reported [7]. However, reports on the influence of Gln in brain of patients with dementia are controversial. One review paper has reported an association of high Gln concentration in plasma with lower cognitive function [8]. Moreover, it has been reported that high Gln concentrations in brain are associated with the levels of consciousness disturbance in patients with hepatic encephalopathy [9]. Furthermore, high plasma Gln concentrations have been identified in patients with liver failure [10]. Given those previous reports, we hypothesized that the Gln concentrations in plasma and brain could be associated. Investigating the association is of importance, since Gln is currently part of clinical nutrition supplementation protocols and recommended for immune suppressed individuals despite insufficient information about the influence on brain Gln concentrations [11]. While an increasing number of metabolomics studies, using mass spectrometry analysis or magnetic resonance spectroscopy (MRS), have focused on the relation between plasma/brain metabolites and symptoms in neuropsychiatric/neurological diseases, none of them have identified a correlation between plasma and brain metabolite concentrations.

While a previous study reported an increase in the cerebral Gln [7] concentrations upon Gln infusion into the blood, another study reported that there was no association between the concentrations of this metabolite in brain and plasma [12]. In the latter study, Gln concentrations in plasma and brain were measured on different days, with the study design therefore possibly affecting the association between Gln concentrations in plasma and brain. Moreover, the authors could not measure Gln independently from Glu; thus, they investigated the association between the amount of Gln in plasma and the sum of Glu and Gln (Glx) in brain measured by MRS. 

Glu is an excitatory neurotransmitter that exists more abundantly in brain than Gln. Glu is the main component of umami substances [13] and thus also exists in plasma. While there have been some concerns that too much monosodium Glu (MSG) intake may be harmful for the brain [14], it is considered that Glu does not enter the brain due to the blood brain barrier (BBB). Given the frequent use of MSG as umami substances in daily life, it is of importance to clarify the relation between plasma and brain concentrations of Glu. 

In this study, we aimed to investigate the association between Glu–Gln concentrations in plasma with those in brain by liquid chromatography–mass spectrometry (LC/MS) and MRS, respectively. For the MRS volume of interest (VOI), two different brain regions were scanned, namely, the posterior cingulate cortex (PCC) and the cerebellum (Cbll). PCC is an important hub of the default mode network and is associated with cognitive impairment in brain disorders, such as Alzheimer’s disease [15]. Cbll has a different histology from other brain regions and is also associated with several brain disorders [16] as well as several brain functions [17].

## 2. Materials and Methods 

### 2.1. Participants

This study included 10 males and 10 females, with a mean ± standard deviation (SD) age of 22.6 ± 1.8 and 23.0 ± 2.3 years, respectively. The study subjects reported neither drug/alcohol abuse nor any mental illness. Written informed consent was obtained from all subjects, and the study protocol was approved by the Ethics and Radiation Safety Committee of the National Institute of Radiological Sciences.

### 2.2. MRS Acquisition

**Figure 1:**
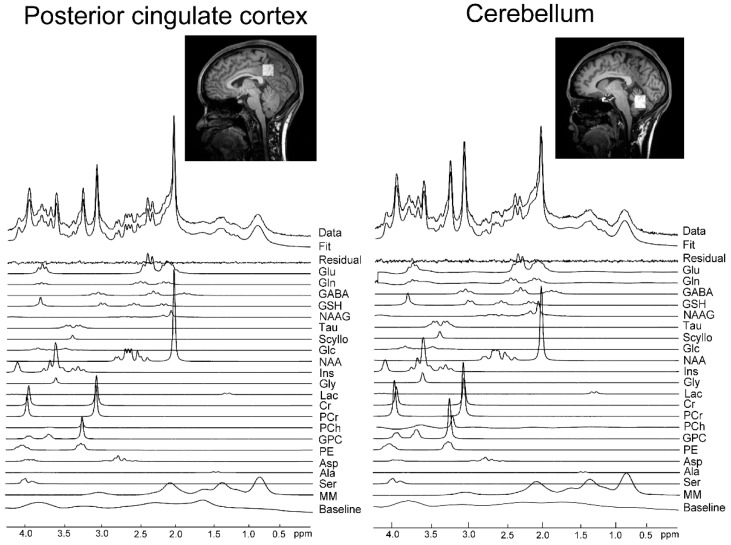
Representative spectrum in posterior cingulate cortex (PCC) and cerebellum (Cbll). Volumes of interest (VOIs) of MRS were localized at PCC and Cbll with the following voxel volumes: VOI 20 × 20 × 20 mm^2^ for PCC, and 25 × 25 × 20 mm^2^ for Cbll. A representative magnetic resonance (MR) spectrum acquired with the SPECIAL sequence at 3T (TE/TR = 8.5/3000 ms, number of averages = 128), the corresponding LCModel spectral fit, fit residual, macromolecules (MM), baseline and individual metabolite fits including glutamate (Glu) and glutamine (Gln).

A 3T scanner (Siemens MAGNETOM Verio, Erlangen, Germany) with a 32-channel receiving head coil was used for magnetic resonance imaging and MRS. Anatomical images were acquired by magnetization-prepared rapid gradient-echo and utilized for determining the voxels of interest. The VOIs were localized at PCC and Cbll (Figure 1) so that each VOI was placed in the PCC or cerebellar vermis/hemispheres, and measured by a short echo time (TE) spin-echo full-intensity acquired localized single voxel spectroscopy (SPECIAL) sequence [18,19,20] with the following parameters: TE = 8.5 ms, repetition time (TR) = 3000 ms, 128 averages, and VOI = 20 × 20 × 20 mm^2^ for PCC and 25 × 25 × 20 mm^2^ for Cbll. After 3D Shim (Syngo MR version for B17, Siemens, Erlangen, Germany) was performed, manual shimming was performed so that the linewidth of the water spectrum in magnitude mode became smaller than 20 Hz. Outer volume suppression (OVS) [21] and water suppression with variable-pulse power and optimized relaxation delays (VAPOR) [21] were applied prior to the SPECIAL localization sequence. MRS was sequentially performed twice, with a 60-min interval, in all participants. To aim for identical localizations of both measurements, the screenshot images of the first MRS VOI placements were saved and utilized as a reference for the second MRS measurements. Tissue composition inside the VOI was calculated based on the segmentation of 3D T1-weighted images using Gannet3.0 [22]. Water concentrations, used in LCModel analysis, were calculated based on the volume fractions of white matter (WM), grey matter (GM) and cerebrospinal fluid (CSF), assuming water concentrations of WM, GM and CSF of 35,880, 43,300 and 55,556 mM, respectively. Metabolite concentrations were then divided by the fraction of WM and GM to correct for CSF inside the VOI, since metabolites are mainly present in WM and GM [20]. The signal-to-noise ratio (SNR) was obtained using N-acetylaspartate (NAA) peak height at 2.01 ppm divided by standard deviation (SD) of noise. For all spectra, LCModel quantification was performed on a spectral window between 0.2 and 4.2 ppm. Macromolecules (MM) were fit using LCModel’s default parametrized MM resonances, and the default LCModel baseline parameters were used.

### 2.3. MRS Data Analysis

A weighted combination of receiver channels was used, followed by removal of motion corrupted averages, spectral registration for frequency and phase drift correction, and alignment of subspectra prior to subtraction; this was performed with MATLAB (The Mathworks, Natick, MA, USA) using the FID-A toolkit prior to signal averaging and data analysis [23]. The linear combination (LC) model [24] with a basic set including 21 simulated metabolite spectra containing alanine (Ala), aspartate (Asp), phosphocholine (PCh), creatine (Cr), phosphocreatine (PCr), γ-aminobutyric acid (GABA), Gln, Glu, glutathione (GSH), glycine (Gly), myo-inositol (mI), lactate (Lac), NAA, scyllo-inositol (Scyllo), taurine (Tau), glucose (Glc), N-acetylaspartylglutamate (NAAG), glycerophosphocholine (GPC), phosphorylethanolamine (PE), Serine (Ser) and macromolecule signals was used to analyze MRS data. In the outcome measures, individual neurochemical concentrations were acquired by performing partial volume correction as indicated above. Moreover, the total Cr (tCr; Cr + PCr) was utilized for normalization, since tCr is widely used as an internal reference in human studies [25].

### 2.4. Biochemical Analysis, LC/MS

All participants had breakfast before 9 am and then fasted for at least 4 h. Blood samples were collected at 2 pm after the first MRS scan and stored in tubes containing disodium ethylenediaminetetraacetate (2Na·EDTA). Blood could not be collected from one female participant due to a technical issue, resulting in the retrieval of 19 blood samples (from 10 males and 9 females) in total. Plasma was prepared by centrifuging the samples at 3000 rpm at 4 °C for 15 min and then stored at −80 °C until analysis. The plasma amino acid measurements were conducted at a commercial laboratory (SRL Co., Ltd., Tokyo, Japan) according to established procedures [26,27]. The plasma samples were deproteinized using acetonitrile at a final concentration of 80.0% before the measurements were performed. The plasma concentrations of 39 human amino acids were measured using high-performance liquid chromatography and electrospray ionization mass spectrometry (HPLC–ESI–MS) (Hitachi High-Technologies, Ibaraki, Japan), followed by pre-column derivatization using previously described analytical methods [26,27,28], and Glu and Gln sample concentrations were used for subsequent analyses. 

### 2.5. Statistics

The results were presented as mean ± SD. Spearman’s correlation coefficient was used to examine the association between Gln and Glu concentrations in brain and those in plasma, with Bonferroni-adjusted *p*-value < 0.025 (for Gln and Glu) using IBM SPSS 20 (IBM Corp, Armonk, NY, USA).

## 3. Results

### 3.1. Spectral Quality Assessment for MRS in PCC and Cbll

In both PCC and Cbll, 8 metabolites (tCr, GABA, Gln, Glu, GSH, mI, tNAA (NAA+NAAG), tCho (GPC + PCh)) were measured by Cramér–Rao lower bound (CRLB) <20% (Table 1 and Table S1). Among the 8 metabolites, this study focused on Glu and Gln. Typical spectra and VOIs are shown in Figure 1. The correlation coefficient values of Glu and Gln (LCModel output) for each brain region, in PCC and Cbll, were 0.12 ± 0.05 and 0.13 ± 0.08, respectively. In the first MRS session, spectral SNR and linewidth (LCModel output) were 59.6 ± 7.3, 0.030 ± 0.005 ppm for PCC and 55.3 ± 7.9, 0.039 ± 0.010 ppm for Cbll, respectively. In the second MRS session, spectral SNR and linewidth (LCModel output) were 61.6 ± 7.5, 0.029 ± 0.006 ppm for PCC and 54.9 ± 9.4, 0.038 ± 0.007 ppm for Cbll, respectively. Tissue compositions inside the MRS voxel were measured and used for partial volume correction. 

### 3.2. Test–Retest Reproducibility of MRS Measurements

The test–retest reproducibility of MRS measurements was investigated for Glu, Gln, Glu/tCr, Gln/tCr and tCr in both PCC and Cbll. Across the repeated measurements, the percent coefficient of variance (%CoV), which is defined as the ratio of SD to the mean, was calculated. The results are summarized in Table 2. The %CoV of tCr varied little in this study, which is the rationale for its use as a reference for both Gln and Glu. The ratios of tissue compositions are also shown in Table 3 to provide information about the reproducibility of voxel placements.

### 3.3. Correlations between Glu–Gln Concentrations in Plasma and Brain

**Figure 2:**
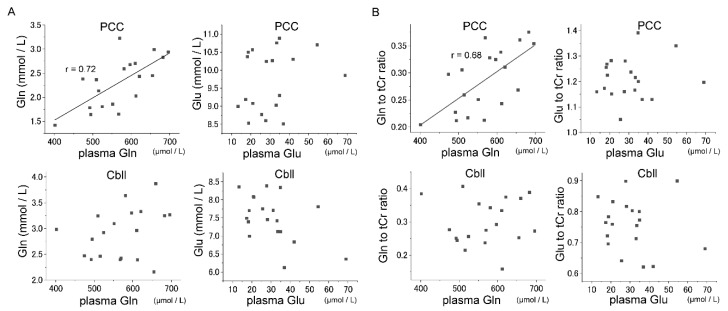
Correlations between plasma glutamate–glutamine (Glu-Gln) and cerebral Glu-Gln. Significant positive correlations between Gln concentrations in plasma and PCC (mean of two measurements) were found (*p* < 0.01, *r* = 0.72). No significant correlation was noted between Gln in plasma and Cbll. Glu did not show any significant correlations between brain and plasma (**A**). Significant positive correlations were found between Gln in plasma and the Gln to tCr ratio (mean of two measurements) in PCC (*p* < 0.01, *r* = 0.68). No other correlations were found (**B**).

The association between Glu and Gln concentrations in brain and that in plasma were investigated, and significant positive correlations were observed between Gln concentrations in PCC and Gln levels in plasma (*p* < 0.025, *r* = 0.72), which is in agreement with the correlation between Gln/tCr and Gln levels in plasma (*p* < 0.025, *r* = 0.68) (Figure 2). A significant correlation between brain and plasma was not observed in the case of Glu. No significant correlation was observed between Gln in plasma and Gln/tCr in Cbll. Considering the lower trend of %CoV observed in PCC than in Cbll for Gln measurements, the absence of a significant correlation in Cbll may also be attributed to the lower reproducibility in Cbll than in PCC. Because Glx (the sum of Glu and Gln) has frequently been used in previous clinical studies—particularly using a 1.5T magnet that cannot appropriately distinguish between Glu and Gln—the association between Glx and the sum of Glu and Gln in plasma was investigated. No significant correlations between Glx concentrations in plasma and brain were observed (data not shown).

## 4. Discussion

In the present study, the glutamatergic metabolite (Gln and Glu) concentrations in plasma and brain were investigated along with their potential associations. Two different brain regions were scanned, namely, PCC and Cbll. PCC is considered a hub of the default mode network and an important region associated with the pathophysiology of Alzheimer’s disease [15,29]. A previous study demonstrated the association between hypometabolism in PCC and neocortical tau protein accumulation and cognitive impairment [30], suggesting that PCC is an important region for the pathomechanism of Alzheimer’s disease. On the other hand, Cbll has a different histology from other brain regions and is frequently utilized as a reference region because of its distinct physiological characteristics. It is also acknowledged as being a more challenging target for MRS because of its magnetic field inhomogeneity as well as moderate distance from the head coil, leading to lower MRS signals.

In this study, a positive correlation was observed between Gln concentrations in plasma and those in PCC. In a previous MRS study of the association between Gln concentrations in plasma and brain, the authors did not find a significant correlation between the two metabolites [12]. In their work, the time lag between measurements may have affected the obtained results, as blood sampling and MRS acquisition were performed on different days. The positive correlation between Gln concentrations observed in the present study may be partially attributable to the consistent schedule of blood sampling and the brain metabolite measurement for LC/MS and MRS, respectively. The strong association between Gln concentrations in plasma and brain corresponds with the findings of a previous study that demonstrated a significant role of Gln transporters in brain and plasma, leading to a significant association between the Gln concentrations in plasma and those in the extracellular brain space [31]. Because Gln is the most abundant amino acid in blood, Gln concentrations in brain may be influenced by Gln in plasma [32]. This assumption seems reasonable, given that Gln concentrations in plasma are the highest among amino acids, and Gln concentrations in brain are comparable to those in plasma (Figure S1). This insight would be of importance when considering therapeutic strategies against hepatic encephalopathy and liver failure, since Gln concentrations in brain [9] and plasma [10], respectively, are elevated in those diseases, thus leading to slower clearance of Gln from brain to plasma [33]. It may also be important to consider meal timing and its condition for MRS examination because Gln concentration in plasma is influenced by food intake [34]. Furthermore, Glx—the sum of Glu and Gln—is frequently used in clinical studies because it is difficult to separate Gln from Glu at 1.5T. Given the influence of Gln in plasma on Gln in brain, caution should be exercised when interpreting Glx alterations. Indeed, a previous study reported that Glx did not significantly correlate with other clinical parameters, whereas Glu did [35].

While a positive correlation was observed between Gln concentrations in PCC and plasma, this was not the case for Gln concentrations in Cbll and plasma. While Gln CRLB in Cbll was not significantly higher than that in PCC, the test–retest reliability data showed the %CoV of Gln measurement to be higher in Cbll than in PCC (Table 2). Thus, the lower measurement precision of Gln in Cbll may be attributable to differences between the two regions, which might be expected because Cbll has higher susceptibility effects and lower SNR due to its location. This could be tested by using a stronger magnet such as 7T in the future. 

Some portion of brain Gln is synthesized in astrocytes via glutamine synthetase, an enzyme that plays an essential role in the metabolism of nitrogen by catalyzing the condensation of Glu and ammonia, after the reuptake of Glu into astrocytes [36]. Gln in astrocytes is then released into blood via the Gln transporter at BBB. Gln concentrations are similar in plasma and CSF, which is an exception among all amino acids [37]. In this study, the positive correlation observed between Gln concentrations in plasma and brain (PCC) may be an important finding with respect to diagnostic and therapeutic strategies for hepatic encephalopathy, the pathomechanism of which remains unclear. Indeed, it has been reported that Gln concentrations in plasma of patients with liver failure were higher in line with their degree of liver failure severity [10]. Moreover, there are increasing lines of evidence that Gln in brain may malfunction in patients with hepatic encephalopathy [33]. Some studies have claimed that the high brain Gln in patients with hepatic encephalopathy is due to increased ammonia in blood, since ammonia is the precursor of Gln, and then high ammonia levels are transferred to synthesize Gln in brain [38]. Given the association between Gln in plasma and brain (PCC), it is also conceivable that high plasma Gln concentrations in patients with liver failure may be attributable to high Gln concentrations in brain due to the transportability between plasma and brain. This insight should be important for developing new therapeutic strategies against hepatic encephalopathy. 

In Alzheimer’s disease, several reports have suggested an association between Gln and brain pathomechanism [39,40]. While Madeira et al., reported that Gln concentrations in CSF of patients with Alzheimer’s disease increased, some controversial reports claimed the opposite [41,42]. To interpret these results, it is important to clarify the association between Glu–Gln concentration in plasma and brain also in terms of pathological conditions.

The regulation of Glu transport by BBB is stricter than that of Gln transport [1], which is justified by the insignificant correlation observed for Glu in this study. Given that Glu in plasma is apparently lower than that in brain (Figure S1) and Glu transport is strictly regulated by BBB, it is hardly likely that MSG intake might cause neurological symptoms. The Glu–Gln cycle is associated with Glu concentration, which is a crucial excitatory neurotransmitter in brain. While reuptake of Glu into astrocytes is a fundamental function in the homeostasis of neurotransmission [43], uptake impairments may cause neuronal toxicities elicited by Glu. While no association was observed between Glu concentrations in plasma and brain in our study, few previous clinical studies have reported reduction in Glu concentration in plasma of patients. In Alzheimer’s disease, BBB becomes impaired with disease progression [44]. Thus, there is a possibility that associations between brain and plasma metabolite concentrations vary depending on brain pathological conditions.

Since all subjects in this work were in their twenties, their tCr levels did not vary significantly (Figure S2), which is the rationale for using tCr as an internal reference. The consistent results regarding the correlations between Gln concentrations in PCC and plasma in both evaluations may suggest that the correlations were not just by chance.

There are a few limitations in this study. First, since we did not investigate patients in this study, we cannot conclude that the association between Gln in plasma and brain exists in other pathological conditions. Given the previous report about high Gln levels in brains of patients with hepatic encephalopathy [33], it can be assumed that there are high Gln levels in plasma and brain in such patients. Thus, it is important to investigate if the associations are maintained in patients with high Gln concentrations in plasma and brain. Second, since we focused on the relation of Glu–Gln in plasma and brain, the inter-relationship between Glu–Gln and other amino acids was not investigated. During periods of hyperammonaemia when increased Gln concentrations are observed (e.g., in patients with hepatic encephalopathy or those with urea cycle disorders), the increase in Gln parallels the decrease in concentration of leucine, isoleucine and valine. Thus, it will be of importance to consider the relation between Glu–Gln and those branched amino acids when investigating the association in the pathological condition in the future.

## 5. Conclusions

In summary, this study demonstrated a positive correlation between Gln concentrations in PCC and plasma. Despite the smaller %CoV in Glu measurements than in Gln measurements, Glu in PCC did not show a positive correlation with that in plasma, which may be due to the more tightly regulated transport by BBB than in the case of Gln. The present work suggests that Gln and Glu are differently transported between brain and plasma. Given the importance of Gln in hepatic encephalopathy and PCC in brain function, the insight gained in this work will be valuable for the investigation of peripheral-CNS pathological biomarkers and the development of therapeutic strategies for brain disorders such as hepatic encephalopathy and Alzheimer’s disease.

## Figures and Tables

**Table 1:** Cramér–Rao lower bound (CRLB) in PCC and Cbll.

	PCC		Cbll	
Metabolites	Mean (%)	SD	Mean (%)	SD
tCr	1.5	0.5	1.3	0.5
GABA	10.7	1.1	9.7	2.2
Gln	10.9	1.6	9.3	1.8
Glu	3.1	0.3	4	0.7
GSH	6.2	0.7	5.8	1.2
mI	4.4	0.7	4.1	0.7
tNAA	1.5	0.5	1.7	0.5
tCho	3.3	0.5	2.4	0.5

Sum of creatine and phosphocreatine (tCr), posterior cingulate cortex (PCC), cerebellum (Cbll), γ-aminobutyric acid (GABA), glutamine (Gln), glutamate (Glu), glutathione (GSH), myo-inositol (mI), sum of N-acetylaspartate and N-acetylaspartylglutamate (tNAA), sum of glycerophosphocholine and phosphocholine (tCho).

**Table 2:** Test–retest reproducibility (percent coefficient of variance, %CoV) of MRS measurements.

	PCC	Cbll
	Mean (%)	Mean (%)
corrected Gln	9.4	14.7
corrected Glu	5.0	8.7
corrected tCr	3.5	3.7
Gln/tCr	7.6	15.2
Glu/tCr	2.3	6.2

**Table 3:** Tissue compositions of VOIs.

	PCC 1st		PCC 2nd		Cbll 1st		Cbll 2nd	
	Mean (%)	SD	Mean (%)	SD	Mean (%)	SD	Mean (%)	SD
GM	75.6	3.1	75.9	3.2	67.0	3.4	67.0	3.8
WM	12.9	2.9	13.7	2.4	29.2	3.9	30.0	4.2
CSF	11.6	3.5	10.4	3.3	3.9	2.8	3.0	2.1

grey matter (GM), white matter (WM), cerebrospinal fluid (CSF).

## Supplementary Materials

The following are available online at https://www.mdpi.com/2072-6643/11/7/1649/s1, Figure S1: Concentrations of Glutamine and Glutamate in Plasma and Brain; Figure S2: tCr Concentrations in PCC and Cbll of Two Measurements; Table S1: Cramér–Rao Lower Bound (CRLB) in PCC and Cbll.

## Abbreviations

MRS	magnetic resonance spectroscopy
LC/MS	liquid chromatography-mass spectrometry
SPECIAL	short-TE spin-echo full-intensity acquired localized single voxel spectroscopy
Gln	glutamine
Glu	glutamate
PCC	posterior cingulate cortex
Cbll	cerebellum
Glx	sum of Glu and Gln
MSG	monosodium glutamate
BBB	blood brain barrier
VOI	volume of interest
OVS	outer volume suppression
VAPOR	variable-pulse power and optimized relaxation delays
%CoV	percent coefficient of variance
CSF	cerebrospinal fluid
WM	white matter
GM	grey matter
SNR	signal-to-noise ratio
NAA	N-acetylaspartate
MM	macromolecules
Ala	alanine
Asp	aspartate
PCh	phosphocholine
Cr	creatine
PCr	phosphocreatine
GABA	γ-aminobutyric acid
GSH	glutathione
Gly	glycine
mI	myo-inositol
Lac	lactate
Scyllo	scyllo-inositol
Tau	taurine
Glc	glucose
NAAG	N-acetylaspartylglutamate
GPC	glycerophosphocholine
PE	phosphorylethanolamine
Ser	serine
2Na·EDTA	disodium ethylenediaminetetraacetate
HPLC-ESI-MS	high-performance liquid chromatography and electrospray ionization mass spectrometry
tCr	sum of Cr and PCr
tNAA	sum of NAA and NAAG
tCho	sum of GPC and PCh
CRLB	Cramér–Rao lower bound
