# Supplementary material for: Association between Brain and Plasma Glutamine Levels in Healthy Young Subjects Investigated by MRS and LC/MS
Source: Nutrients. 2019 Jul 19;11(7):1649. doi: 10.3390/nu11071649 (PMC6682979; doi:10.3390/nu11071649)
Supplement: Supplementary file 1 [file nutrients-11-01649-s001.pdf]

A

PCC		tCr	GABA	Gln	Glu	GSH	mI	tNAA	tCho
1st	Male 1	1	10	10	3	6	4	2	3
	Male 2	2	12	12	4	6	6	2	4
	Male 3	1	11	11	3	6	4	2	3
	Male 4	2	14	11	3	6	5	2	3
	Male 5	2	10	10	3	6	4	1	3
	Male 6	2	10	10	3	7	4	2	3
	Male 7	2	11	10	3	6	4	1	3
	Male 8	1	11	12	3	6	4	1	3
	Male 9	2	10	8	3	6	5	2	4
	Male 10	2	10	11	3	7	4	2	4
	Female 1	2	12	11	3	6	5	2	3
	Female 2	1	9	11	3	6	4	1	3
	Female 3	2	11	14	3	6	5	2	4
	Female 4	2	11	14	3	7	5	2	4
	Female 5	1	11	13	3	6	4	1	3
	Female 6	2	10	9	3	9	7	2	4
	Female 7	1	9	9	3	5	4	1	3
	Female 8	2	11	9	3	6	4	2	4
	Female 9	1	10	11	2	5	3	1	2
	Female 10	2	10	9	3	6	4	1	3
2nd	Male 1	1	10	11	3	6	4	1	3
	Male 2	2	11	12	3	7	5	2	4
	Male 3	1	12	13	3	6	4	1	3
	Male 4	2	10	10	4	5	5	1	4
	Male 5	2	10	10	3	6	4	1	3
	Male 6	2	10	10	3	7	4	2	3
	Male 7	2	12	9	3	6	5	2	3
	Male 8	2	13	11	3	7	4	2	3
	Male 9	1	11	9	3	6	4	2	3
	Male 10	1	9	11	3	7	4	1	3
	Female 1	2	12	10	3	7	4	2	3
	Female 2	1	11	14	3	5	4	1	3
	Female 3	1	11	13	3	6	4	1	4
	Female 4	1	10	13	3	6	4	1	3
	Female 5	1	9	13	3	5	4	1	3
	Female 6	1	9	9	3	6	4	1	4
	Female 7	2	12	10	4	6	5	1	4
	Female 8	1	10	10	3	6	4	2	3
	Female 9	1	10	12	3	6	4	1	3
	Female 10	1	11	10	3	6	5	1	4
	mean (SD)	1.5 (0.5)	10.7 (1.1)	10.9 (1.6)	3.1 (0.3)	6.2 (0.7)	4.4 (0.7)	1.5 (0.5)	3.3 (0.5)

B

Cbll		tCr	GABA	Gln	Glu	GSH	mI	tNAA	tCho
1st	Male 1	1	10	12	4	5	4	2	2
	Male 2	2	8	8	4	6	5	2	3
	Male 3	1	8	8	3	5	4	1	2
	Male 4	2	11	12	6	8	6	3	4
	Male 5	1	10	8	3	5	4	1	2
	Male 6	1	11	10	4	6	4	2	2
	Male 7	1	9	8	4	6	4	2	2
	Male 8	1	7	8	3	4	3	1	2
	Male 9	1	9	7	4	7	4	2	2
	Male 10	2	8	10	5	6	6	2	3
	Female 1	2	9	11	4	6	4	2	3
	Female 2	2	9	9	4	5	4	2	3
	Female 3	1	9	9	3	5	4	1	2
	Female 4	2	10	11	4	6	4	2	3
	Female 5	1	14	9	4	5	3	1	2
	Female 6	1	9	7	4	5	4	1	2
	Female 7	2	8	8	4	5	5	2	3
	Female 8	2	10	10	5	10	3	2	3
	Female 9	1	16	10	4	6	4	1	3
	Female 10	1	9	9	3	5	4	1	2
2nd	Male 1	1	10	10	4	6	4	2	2
	Male 2	1	9	9	4	5	3	1	2
	Male 3	1	15	16	4	6	4	2	2
	Male 4	1	9	8	5	5	4	2	2
	Male 5	1	9	8	4	5	5	2	2
	Male 6	2	8	12	5	7	5	2	3
	Male 7	1	8	8	3	5	4	1	2
	Male 8	1	8	8	4	5	4	2	2
	Male 9	1	9	9	3	5	4	1	2
	Male 10	1	8	11	4	6	3	1	2
	Female 1	2	9	8	5	6	4	2	3
	Female 2	1	9	10	4	5	4	1	2
	Female 3	1	11	10	4	6	4	2	2
	Female 4	1	8	8	4	5	4	1	3
	Female 5	1	9	12	4	6	4	2	2
	Female 6	1	10	8	4	6	4	2	2
	Female 7	2	17	9	4	10	6	2	3
	Female 8	1	10	7	4	5	4	2	2
	Female 9	1	7	9	3	5	4	2	2
	Female 10	1	10	9	3	5	4	2	2
	mean (SD)	1.3 (0.5)	9.7 (2.2)	9.3 (1.8)	4.0 (0.7)	5.8 (1.2)	4.1 (0.7)	1.7 (0.5)	2.4 (0.5)

Supplemental Table S1. Cramér–Rao Lower bound (CRLB) in PCC and Cbll.

All CRLB values of each metabolite, which had the mean CRLB less than 15%, were demonstrated (A. PCC, B. Cbll).

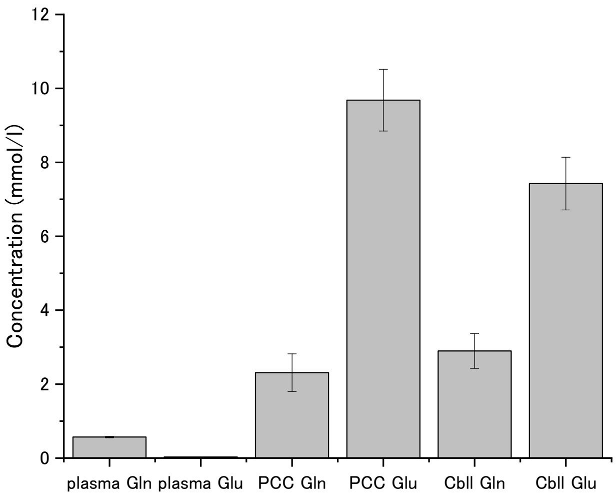

Supplemental Figure S1. Concentrations of Glutamine and Glutamate in plasma and brain.

Concentrations of Glutamine and Glutamate in plasma and brain (PCC and Cbll) are demonstrated. While concentrations of Gln in plasma and brain are in the same order, the differences of Glu concentrations in plasma and brain are huge, indicating that the transportabilities of Gln and Glu are different between plasma and brain.

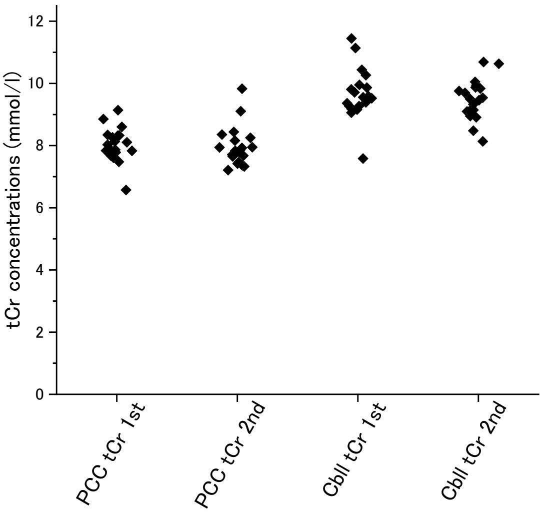

Supplemental Figure S2. tCr concentrations in PCC and Cbll of two measurements.

Each concentration of tCr in all measurements were shown.
